# Prevalence, risk factors, and prognosis of peritoneal metastasis from breast cancer

**DOI:** 10.1186/s40064-015-1449-x

**Published:** 2015-11-10

**Authors:** Serena Bertozzi, Ambrogio P. Londero, Carla Cedolini, Alessandro Uzzau, Luca Seriau, Sergio Bernardi, Stefano Bacchetti, Enrico Maria Pasqual, Andrea Risaliti

**Affiliations:** Clinic of Surgery, AOU “Santa Maria della Misericordia”, DISM, DSMB, University of Udine, Piazzale Santa Maria della Misericordia 15, 33100 Udine, UD Italy; Surgical Oncology Department, IRCSS CRO, Via Franco Gallini, 2, 33081 Aviano, PN Italy; Unit of Obstetrics and Gynecology, S Polo Hospital, via Galvani 1, 34074 Monfalcone, GO Italy; Unit of Surgery, Hospital of Latisana, via Sabbionera 45, 33053 Latisana, UD Italy

**Keywords:** Breast cancer, Distant metastasis, peritoneal metastasis, Peritoneal carcinomatosis, Overall survival

## Abstract

Peritoneal metastasis from breast cancer is a serious and deadly condition only limited considered in the literature. Our aim was to study prevalence, risk factors, and prognosis of breast cancer peritoneal metastasis. We retrospectively analyzed 3096 women with a diagnosis of invasive breast cancer. We took into consideration presence and localization of breast cancer distant metastasis as well as the possible risk factors and survival from the diagnosis of the breast cancer metastasis. The prevalence of breast cancer peritoneal metastases was 0.7 % (22/3096), representing the 7.6 % (22/289) of women affected by distant metastases. Moreover, independent risk factors for breast cancer peritoneal metastases resulted high grading, lobular invasive histology, and advanced T and N stage at diagnosis. Overall survival after metastasis diagnosis was shorter in women affected by peritoneal metastases or brain metastases in comparison to other metastatic women. Breast cancer peritoneal metastases were uncommon but not rare events with a poor prognosis after standard treatments.

## Background

Metastatic breast cancer represents an important challenge for breast specialists, and its incidence has not changed during the last decades, while we have assisted to a progressive reduction of locally advanced breast cancers and a consensual increase of early breast ones, thanks to the introduction of an organized mammographic screening in our region since 2005 (Driul et al. 2013; Cedolini et al. 2014a). A first explanation may be that distant metastasis by haematogenous way do not depend on tumor size neither on lymph node involvement, which is usually expression of metastatization by lymphatic way. Secondly, groups of patients who more frequently present distant metastasis from breast cancer do not usually represent a screening target, such as young pre-menopausal women.

Typical breast cancer metastatization sites are, in order of frequency, bones, liver, lungs and brain, but many other secondary localizations have been described in the literature, including the peritoneal cavity (Sheen-Chen et al. 2008). In fact, although peritoneal carcinomatosis usually affects patients with solid intra-abdominal cancers, including those originated by the gastrointestinal tract and those originating by the female reproductive system (Pasqual et al. 2014; Pasqual et al. 2012), it may also derive from any other solid tumor of the human body.

Peritoneal carcinomatosis represents a lifethreating condition, with a very high mortality rate (Pasqual et al. 2014; Pasqual et al. 2012), which was historically subjected to systemic chemotherapy, exclusively with a palliative intent. Anyway, during the last two decades new loco-regional integrated treatments have been established, in order to treat patients affected by peritoneal carcinomatosis, in some selected cases even with curative intent (Pasqual et al. 2014; Pasqual et al. 2012). In particular, surgical cytoreduction combinated to intraperitoneal chemotherapy, which was firstly introduced by Sugarbaker in 1995 showed very encouraging results in improving both overall and disease-free survival of this group of patients (Sugarbaker 1995; Roviello et al. 2011).

Our study aims to determine the prevalence and risk factors for peritoneal carcinomatosis among breast cancer patients, and to compare their prognosis with that of other secondaries sites.

## Methods

We collected retrospective data about 3096 women operated at their breast for invasive breast cancer. We focused on patients operated between 2001 and 2010, in order to have at least 4 years of follow up. We excluded women affected by intraductal neoplasia or benign breast diseases. This retrospective chart review study was performed according to the Declaration of Helsinki and approved by the Internal Review Board. In addition, this study regarding consent for processing data used in this retrospective analysis follows the dictates of the general authorisation to process personal data for scientific research purposes by the Italian Data Protection Authority.

We took into consideration patients characteristics as follows: age and BMI (body mass index) at the time of diagnosis, familial history of breast cancer, eventual menopausal status and estro-proestinic drug assumption. Among tumor characteristics we considered: histological type, TNM classification and stage, eventual loco-regional extra-axillary lymph node involvement (internal mammary chain and subclavear), nuclear grading, Mib1/Ki-67 proliferation index, estrogen and progesteron receptors expression, Her2/neu status, and molecular subtypes. Moreover, we included other microscopical histological characteristics which are included in the more recent classification purposed by Veronesi and colleagues (Arnone et al. 2010) as previously described (Cedolini et al. 2014a, b, c; Bertozzi et al. 2013; Bernardi et al. 2012a, b), including multifocality/multicentricity, extensive intraductal component, perivascular invasion, peritumoral inflammation, lymph node extra-capsular invasion, and presence of bunched lymph nodes. Finally, we took into consideration also the therapeutic management: type of intervention on the breast (conservative breast surgery or mastectomy) and the axilla (sentinel lymph node biopsy or complete lymph node axillary dissection), eventual neoadjuvant or adjuvant therapies (chemotherapy, hormonal therapy, biological drugs, radiation therapy).

Then, we identified the group of women affected by peritoneal metastasis from breast cancer, and compared them with non metastatic patients and those with distant metastasis other than peritoneum. In particular, peritoneal carcinomatosis was suspected in any women presenting with ascitis, or with a documented increase in CA125, or with unexplained anorexia and weigh loss of recent onset. Thereafter, in any case abdominal CT scan was performed, with or without additional FDG-PET/CT, in order to confirm peritoneal involvement. By the presence of ascitis, peritoneal fluid cytology was also performed.

Data were analyzed using R (version 3.1.0: http://www.R-project.org/), considering significant p < 0.05. Distribution normality of each variable was assessed by Kolmogorov–Smirnov test. The following statistical test were used when appropriate: *t* test and Wilcoxon test for continuous variables, Chi-square test or Fisher exact test for categorical ones. Univariate and multivariate logistic regression analysis was performed considering as dependent variables the presence of distant metastases or peritoneal metastases. Kaplan–Meier curves were drown to show overall survival, and the differences among the three groups were determined through the Log-rank test. Univariate and multivariate Cox proportional hazards regression models were performed considering the possible factors influencing survival.

## Results

Among the 3096 considered women affected by invasive breast cancer, 289 (9.33 %) presented a distant metastasis at the time of diagnosis or developed a metastasis at a median follow up of 83 months. Only 1.8 % (57/3096) of cases were initially diagnosed as stage IV breast cancer, and thus a minority (19.7 %) of whole metastatic breast cancer population. Moreover, median survival after the diagnosis of metastasis resulted 28 months.

Distant metastases affected the following sites in order of frequency: bones 67.8 % (196/289), liver 47.8 % (138/289), lungs 42.6 % (123/289), distant lymph nodes 27 % (78/289), brain 15.2 % (44/289), peritoneum (peritoneal carcinomatosis) 7.6 % (22/289), and other sites 6.9 % (20/289). Only 29.8 % (86/289) of women had only one metastatic site involved, while in all other cases distant metastases affected more than one site. Moreover, the prevalence of peritoneal metastases was 0.7 % (22/3096).

Mean age at diagnosis of our population was 60.15 years (± 13.03), mean BMI 25.86 kg/m^2^ (±4.93), and 74.9 % of women were in post-menopause. Surgical approach was conservative in the most cases. The majority of breast cancers resulted to be non-special type (e.g. ductal invasive carcinoma) in the 76.3 % (2362/3096) of cases, small sized (T1) in the 72.4 % of cases, and without lymph node involvement (N0) in the 66.4 % of patients. Estrogen receptor was expressed even in the 84.1 % of cases. The more prevalent molecular subtype resulted luminal A in 31.2 % (966/3096) of cases, followed by luminal B in 23.8 % (738/3096) of patients, whereas only a minority were diagnosed as basal-like or Her-enriched subtypes.

Comparing patients with peritoneal, non-peritoneal metastases, and without metastases, we can notice that women who develop peritoneal carcinomatosis (M1: peritoneum) were younger and have a lower BMI (Table 1). Moreover, they usually underwent more aggressive adjuvant treatment, probably due to their unfavorable prognosis at diagnosis, related to bad molecular subtypes, high grading and advanced TNM stage (Table 2). Finally, development of metastasis in the peritoneal cavity happened significantly later than those of any other site: at 5 years follow up have already appeared 77.1 % (95 % C.I. 70.5–82.2 %) of the non-peritoneal metastases while only 60.0 % (95 % C.I. 25.7–78.5 %) of peritoneal metastases have appeared.

**Table 1:** Characteristics of the population and differences among controls (M0) and women affected by peritoneal carcinomatosis (M1: peritoneum) or other site of metastases (M1: other than peritoneum)

	M0	M1	M1	p
		Other than peritoneum	Peritoneum	
Age at diagnosis (years)	60.19 (± 12.92)	60.07 (± 14.2)	56.59 (± 12.52)	NS
BMI (kg/m^2^)	25.89 (± 4.9)	25.86 (± 5.11)	22.42 (± 4.97)	(2, 3)
Tobacco smoke	7.5 % (168/2230)	4.3 % (9/208)	0 % (0/19)	NS
Familial history of breast cancer	36 % (283/787)	33.9 % (21/62)	50 % (1/2)	NS
Use of OC	29.7 % (156/525)	24.5 % (12/49)	0 % (0/2)	NS
Post-menopausal status	79.6 % (2110/2652)	78.1 % (196/251)	70 % (14/20)	NS
First breast surgical treatment				
Breast conserving surgery	62.9 % (1767/2807)	33.7 % (90/267)	54.5 % (12/22)	(1, 3)
Mastectomy	37.1 % (1040/2807)	66.3 % (177/267)	45.5 % (10/22)	(1, 3)
Axilla surgical treatment				
CALND	53.8 % (1511/2807)	90.3 % (241/267)	86.4 % (19/22)	(1,2)
SLNB	42.5 % (1194/2807)	5.6 % (15/267)	9.1 % (2/22)	(1, 2)
None	3.6 % (102/2807)	4.1 % (11/267)	4.5 % (1/22)	NS
Second breast surgical treatment				
None	77 % (1285/1669)	65.9 % (54/82)	81.8 % (9/11)	(1)
Breast conservative surgery	10.7 % (178/1669)	14.6 % (12/82)	0 % (0/11)	NS
Mastectomy	12.3 % (206/1669)	19.5 % (16/82)	18.2 % (2/11)	NS
Other treatments				
Neoadjuvant	5.6 % (158/2807)	15.7 % (42/267)	9.1 % (2/22)	(1)
Adjuvant radiotherapy	58 % (1561/2690)	47.8 % (121/253)	50 % (11/22)	(1)
Adjuvant chemotherapy	39.1 % (1051/2687)	82.9 % (208/251)	86.4 % (19/22)	(1, 2)
Adjuvant hormonal therapy	79.7 % (2140/2684)	64.5 % (162/251)	72.7 % (16/22)	(1)

Significant differences (p < 0.05) between: (1) M0 vs M1: other than peritoneum; (2) M0 vs M1: peritoneum; (3) M1: other than peritoneum vs M1: peritoneum

*NS* non significant

**Table 2:** Tumor characteristics and staging among controls (M0) and women affected by peritoneal carcinomatosis (M1: peritoneum) or other site of metastases (M1: other than peritoneum)

	M0	M1	M1	p
		Other than peritoneum	Peritoneum	
Histological type				
Ductal invasive carcinoma	77.4 % (2173/2807)	66.7 % (178/267)	50 % (11/22)	(1, 2)
Lobular invasive carcinoma	12.1 % (340/2807)	16.5 % (44/267)	36.4 % (8/22)	(1, 2, 3)
Ductal and lobular invasive carcinoma	6.7 % (189/2807)	12.7 % (34/267)	9.1 % (2/22)	(1)
Other invasive carcinoma	3.7 % (105/2807)	4.1 % (11/267)	4.5 % (1/22)	NS
Molecular Subtype				
Luminal A	32.9 % (923/2807)	13.5 % (36/267)	31.8 % (7/22)	(1, 3)
Luminal B	23.2 % (652/2807)	29.6 % (79/267)	31.8 % (7/22)	(1)
Luminal Her	5.5 % (153/2807)	8.2 % (22/267)	9.1 % (2/22)	(1*)
Her enriched	4 % (111/2807)	8.6 % (23/267)	0 % (0/22)	(1)
Basal-like	7.3 % (205/2807)	16.5 % (44/267)	9.1 % (2/22)	(1)
Non described	27.2 % (763/2807)	23.6 % (63/267)	18.2 % (4/22)	NS
Other characteristics of the primary tumor				
ER positives	85.2 % (2284/2680)	71.7 % (180/251)	90.9 % (20/22)	(1, 3*)
PgR positives	73 % (1958/2681)	55.8 % (140/251)	59.1 % (13/22)	(1)
HER2/neu positives	11.8 % (267/2254)	19.4 % (45/232)	9.5 % (2/21)	(1)
Ki-67/Mib-1 >20	33.7 % (671/1994)	61.1 % (113/185)	29.4 % (5/17)	(1, 3)
Comedo-like necrosis	8.7 % (243/2807)	7.9 % (21/267)	0 % (0/22)	NS
Multifocality/multicentricity	20.2 % (567/2807)	22.5 % (60/267)	18.2 % (4/22)	NS
Extensive intraductal component	27.2 % (764/2807)	21.3 % (57/267)	13.6 % (3/22)	(1)
Lymphovascular invasion	15.6 % (439/2807)	26.6 % (71/267)	18.2 % (4/22)	(1)
Peritumoral inflammation	3.3 % (93/2807)	9.4 % (25/267)	0 % (0/22)	(1)
Loco-regional lymph nodes characteristics				
Non-axillary loco-regional lymph nodes	1.7 % (48/2807)	1.5 % (4/267)	0 % (0/22)	NS
Isolated tumor cells	2 % (56/2807)	0.4 % (1/267)	0 % (0/22)	(1**)
Micrometastases	4.9 % (138/2807)	4.1 % (11/267)	4.5 % (1/22)	NS
Extracapsular invasion of lymph node metastasis	7.1 % (198/2807)	28.1 % (75/267)	36.4 % (8/22)	(1, 2)
Bunched axillary lymph nodes	2.1 % (58/2807)	18 % (48/267)	31.8 % (7/22)	(1, 2)
TNM				
T1	75.4 % (2116/2807)	41.2 % (110/267)	68.2 % (15/22)	(1,3)
T2	20.9 % (586/2807)	37.8 % (101/267)	13.6 % (3/22)	(1, 3**)
T3	1.6 % (44/2807)	10.1 % (27/267)	4.5 % (1/22)	(1)
T4	2.2 % (61/2807)	10.9 % (29/267)	13.6 % (3/22)	(1, 2)
N0	69.9 % (1961/2807)	32.6 % (87/267)	36.4 % (8/22)	(1, 2)
N1	21.2 % (594/2807)	23.2 % (62/267)	13.6 % (3/22)	NS
N2	5.3 % (150/2807)	17.2 % (46/267)	9.1 % (2/22)	(1)
N3	3.6 % (102/2807)	27 % (72/267)	40.9 % (9/22)	(1, 2)
Grading				
G1	15.8 % (444/2807)	2.6 % (7/267)	0 % (0/22)	(1, 2)
G2	60.3 % (1693/2807)	49.8 % (133/267)	90.9 % (20/22)	(1, 2, 3)
G3	23.9 % (670/2807)	47.6 % (127/267)	9.1 % (2/22)	(1, 3)

Significant differences (p < 0.05) between: (1) M0 vs M1: other than peritoneum; (2) M0 vs M1: peritoneum; (3) M1: other than peritoneum vs M1: peritoneum

*NS* non significant

Other differences: (1*) p = 0.060; (3*) p = 0.051; (1**) p = 0.058; (3**) p = 0.070

The multivariate logistic regression highlighted the following predisposing factors for breast cancer metastasis: high grading, lobular invasive histotype (including ductal-lobular invasive carcinoma), molecular subtype other than luminal A, advanced TNM stage (including both big tumor size and high lymph node involvement) (Table 3a). Taking into consideration separately stage I, II and III at diagnosis, the multivariate logistic regression confirmed the previous predisposing factors for presence of distant metastases (Table 3b).

**Table 3:** Risk factors for distant metastases (M1) in multivariate logistic regression analysis: (A) considering TNM stage I, II, III, and IV at diagnosis; (B) considering only TNM stage I, II, and III at diagnosis

	OR (C.I. 95 %)	p
(A)		
Grading	1.64 (1.25–2.15)	<0.05
Histological type		
Ductal invasive carcinoma	Referral	1.000
Lobular invasive carcinoma	1.80 (1.21–2.65)	<0.05
Ductal and lobular invasive carcinoma	1.68 (1.07–2.64)	<0.05
Other invasive carcinoma	1.23 (0.60–2.51)	0.570
Molecular subtype		
Luminal A	Referral	1.000
Luminal B	1.86 (1.21–2.87)	<0.05
Luminal Her	2.34 (1.29–4.23)	<0.05
Her enriched	2.20 (1.15–4.22)	<0.05
Basal-like	2.89 (1.69–4.94)	<0.05
Non described	1.87 (1.20–2.93)	<0.05
TNM		
T1	Referral	1.000
T2	1.53 (1.12–2.11)	<0.05
T3	3.87 (2.16–6.92)	<0.05
T4	2.62 (1.50–4.58)	<0.05
N0	Referral	1.000
N1	2.00 (1.41–2.85)	<0.05
N2	4.26 (2.78–6.53)	<0.05
N3	8.81 (5.81–13.37)	<0.05
(B)		
Grading	1.55 (1.15–2.09)	<0.05
Histological type		
Ductal invasive carcinoma	Referral	1.000
Lobular invasive carcinoma	1.96 (1.29–3)	<0.05
Ductal and lobular invasive carcinoma	1.5 (0.9–2.48)	0.118
Other invasive carcinoma	0.79 (0.3–2.07)	0.629
Molecular subtype		
Luminal A	Referral	1.000
Luminal B	2.42 (1.48–3.97)	<0.05
Luminal Her	2.61 (1.33–5.13)	<0.05
Her enriched	2.28 (1.07–4.84)	<0.05
Basal-like	3.66 (2–6.72)	<0.05
Non described	2.26 (1.35–3.78)	<0.05
TNM		
T1	Referral	1.000
T2	1.55 (1.09–2.19)	<0.05
T3	3.12 (1.62–6.03)	<0.05
T4	2.16 (1.13–4.13)	<0.05
N0	Referral	1.000
N1	2.23 (1.51–3.28)	<0.05
N2	4.79 (3–7.63)	<0.05
N3	7.95 (4.96–12.73)	<0.05

Furthermore, by multivariate logistic regression, the following factors resulted to be significantly predictive for the development of peritoneal metastasis: high grading, lobular invasive histotype (including ductal-lobular invasive carcinoma), and advanced TNM stage (including both big tumor size and high lymph node involvement) (Table 4a). Thereafter, considering separately stage I, II and III at diagnosis, the multivariate logistic regression confirmed the same predisposing factors for peritoneal metastatization, with the exception of grading and tumor size (Table 4b).

**Table 4:** Risk factors for peritoneal carcinomatosis (M1: peritoneum) in multivariate logistic regression analysis: (A) considering TNM stage I, II, III, and IV at diagnosis; (B) considering only TNM stage I, II, and III at diagnosis

	OR (C.I. 95 %)	p
(A)		
Grading	1.93 (1.47–2.54)	<0.05
Histological type		
Ductal invasive carcinoma	Referral	1.000
Lobular invasive carcinoma	1.71 (1.13–2.57)	<0.05
Ductal and lobular invasive carcinoma	1.54 (0.95–2.52)	0.083
Other invasive carcinoma	1 (0.4–2.5)	0.999
Peritumoral inflammation	1.74 (1–3.01)	0.050
TNM		
T1	Referral	1.000
T2	1.56 (1.11–2.21)	<0.05
T3	3.26 (1.69–6.28)	<0.05
T4	2.06 (1.07–3.99)	<0.05
N0	Referral	1.000
N1	2.23 (1.52–3.27)	<0.05
N2	4.8 (3.03–7.62)	<0.05
N3	7.64 (4.81–2.12)	<0.05
(B)		
Histological type		
Ductal invasive carcinoma	Referral	1.000
Lobular invasive carcinoma	3.58 (1.09–11.75)	<0.05
Ductal and lobular invasive carcinoma	2.57 (0.51–12.83)	0.251
Other invasive carcinoma	3.67 (0.43–31.53)	0.237
TNM		
T1	Referral	1.000
T2-3-4	0.31 (0.09–1.13)	0.076
N0	Referral	1.000
N1	2.2 (0.52–9.33)	0.287
N2	6.36 (1.15–35.22)	<0.05
N3	17.74 (4.52–69.63)	<0.05

**Fig. 1:**
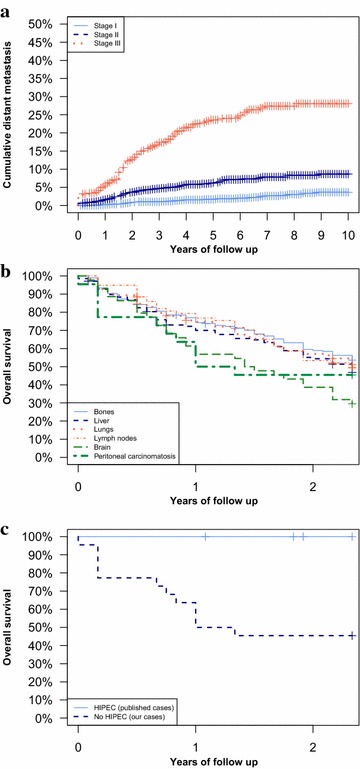
**a** Cumulative distant metastases appearance during the follow-up and TNM stage at diagnosis, **b** 2 years overall survival after the appearance of distant metastases. The difference between women affected by pertitoneal carcinomatosis (M1—peritoneum) and other metastases (excluding brain) was statistically significant (p < 0.05) as well as the difference between women affected by brain metastases and women affected by other metastases (p < 0.05). **c** Two years overall survival after the appearance of peritoneal carcinomatosis (M1—peritoneum) in our population and in cases referred by the literature to be treated by HIPEC procedure (Cardi et al. 2013; Garofalo et al. 2006). We found a significant longer survival in patients treated by HIPEC procedure (p < 0.05)

If we have a look at cumulative metastasis rates, we can see that after 10 years of follow up more than 5 % of breast cancer patients have a distant metastasis diagnosed. In addiction, among stage III patients more than 25 % will develop a breast cancer metastasis during their follow up (Fig. 1a). If we consider overall survival from the date of metastasis diagnosis, brain and peritoneal secondaries resulted to have the worst prognosis (Fig. 1b). In particular, the lower overall survival of peritoneal carcinomatosis becomes statistically significant in comparison with all other metastatic site considering the first 2 years from its diagnosis (p < 0.05), time after which they align with the survival of other metastatic sites. Finally, considering the Cox proportional hazards multivariate regression model analysis, after adjusting data for basal-like molecular subtype and grading, peritoneal carcinomatosis resulted to be an independent risk factor for reduced survival in comparison with other metastatic sites (HR 1.70, 95 % C.I. 1.00–2.90, p < 0.05).

## Discussion

Distant metastasis prevalence among breast cancer patients resulted 9.3 % (289/3096), in the 19.7 % of metastatic cases metastases were synchronous with the primary cancer diagnosis, whereas in the remaining 80.3 % metastases were metachronous after 83 months of median follow up. Peritoneal carcinomatosis had a prevalence of 0.7 % (22/3096), and developed later than all other sites secondaries. By multivariate analysis, high grading, lobular invasive histotype, and advanced TNM stage resulted significantly predictive for peritoneal carcinomatosis. Overall metastasis rate resulted 5 % at 10 years, and even 25 % by considering separately stage III cancers. Median survival after the diagnosis of metastasis resulted 28 months, and was significantly lower for brain and peritoneal secondaries.

The literature did rarely mention peritoneal metastasis from breast cancer, and described peritoneal recurrences after a variable time interval between 5 and 10 years from the diagnosis of primary breast cancer (Eitan et al. 2003; Bigorie et al. 2010; Tserkezoglou et al. 2006; Garg et al. 2013). This late onset might not always be related to a late metastasis development rather than to a late metastasis detection, which results very difficult in the absence of any specific symptom. Moreover, modern imaging techniques resulted scarcely accurate in peritoneal carcinomatosis diagnosis. In fact, CT scan has an important size limit for lesions detection, especially in some critical sites like the small bowel walls (Pasqual et al. 2014; Pasqual et al. 2012), while FDG-PET/CT has a high rate of false positives due to tissue inflammation following medical and radiation therapies, as well as a high rate of false negatives because of metabolic inactivity of dormant neoplastic cells after chemotherapy (Pasqual et al. 2014; Pasqual et al. 2012).

In our population, peritoneal metastases prevalence resulted even 0.7 % (22/3096), and thus just little lower than brain metastasis prevalence. Considering the late onset of peritoneal recurrence in our population, their prevalence may be the result of an increased survival of breast cancer women affected by other sites metastasis thanks to the progressive improvement of medical and radiological therapies, who consequently gain enough time to develop peritoneal metastases. And actually, the most cases of peritoneal involvement by breast cancer (81.8 %) were associated with metastases in other sites.

Focusing on the risk factors for peritoneal metastasis among patients affected by breast cancer, our data highlight a high prevalence of lobular invasive histotype among women who develop peritoneal metastasis, but the literature is very controversial about a histological predisposition. In fact, some Authors described a higher prevalence of invasive lobular carcinoma among patients with peritoneal secondaries than controls (up to 40 %) (Cardi et al. 2013), and an important predisposition of invasive lobular carcinoma to metastasize to any site of the digestive tract (Nazareno et al. 2006; Borst and Ingold 1993). On the other side, some Authors showed a prevalence of invasive ductal carcinoma comparable with that of their general breast cancer population (77 %) (Tuthill et al. 2009; Glass et al. 2007).

Furthermore, some Authors described a higher prevalence of peritoneal carcinomatosis among women with documented mutations of the BrCa genes, which may also be interpreted as a new primary tumor from ovary, fallopian tube or peritoneum itself, all cancers which BrCa mutated patients are also predisposed to (Hewitt et al. 2006). A retrospective study among women with peritoneal metastasis from breast cancer observed a prevalence of peritoneal disease from ovarian cancer or primary peritoneal malignancy of even 74.7 %, while just the remaining 25.3 % of carcinomatosis resulted derived from the previous breast cancer (Garg et al. 2013). In our population, we found a weak association between familial history of breast cancer and peritoneal metastasis, but data lack about both eventual BrCa mutations and peritoneal disease histology, so that we can not completely exclude the possibility of a new primary.

For what concerns the prognosis of metastatic breast cancer, peritoneal secondaries showed a very poor survival, as well as brain ones. During the last decades, the progress in loco-regional integrated treatments allowed a significant improvement in breast metastasis control, or even annihilation. We might cite the development of targeted biological drugs against some specific breast cancer subtypes, the possibility of using bone cement against destructive bone metastasis, the application of interventional radiological techniques against liver secondaries, the minimal invasive and selective surgery against lung lesions, and the use of gamma knife against brain secondaries.

In this perspective, surgical cytoreduction and HIPEC showed encouraging results among selected patients treated in specialized centers. Although nowadays this procedure results standardized only for peritoneal carcinomatosis deriving by cancers of the digestive tract or the female genital system, recently some patients with peritoneal metastasis from breast cancers successfully underwent this treatment (Cardi et al. 2013; Garofalo et al. 2006).

Despite the small number of patients treated with cytoreduction and HIPEC, their survival resulted surely longer than those who underwent surgical debulking without HIPEC and those who were given only systemic chemotherapy. In particular, in the literature patients who underwent surgical debulking resulted to have survivals between 10 and 54 months from the diagnosis of peritoneal carcinomatosis (Eitan et al. 2003; Bigorie et al. 2010; Tserkezoglou et al. 2006; Garg et al. 2013; Ayhan et al. 2005), and thus comparable with those of other metastatic breast cancer women (Altekruse et al. 2010; Jemal et al. 2004), as well as with those affected by stage III ovarian cancer (Eitan et al. 2003; Bigorie et al. 2010; Tserkezoglou et al. 2006; Garg et al. 2013; Moore et al. 2012). Finally, if we compare patients who underwent cytoreduction and HIPEC for breast peritoneal metastasis (data from the literature) (Cardi et al. 2013; Garofalo et al. 2006) with our patients affected by breast cancer peritoneal disease who were simply given palliative systemic chemotherapy, overall survival resulted significantly longer in the first group (Fig. 1c).

In conclusion, the prognosis of peritoneal metastasis from breast cancer resulted very poor. Anyway, we can suggest that in at least the 20 % of them, who presented with isolated peritoneal breast cancer recurrence, surgical cytoreduction and HIPEC might be feasible and effective in improving their overall survival and, most important, their quality of life.

## Abbreviations

BMI: body mass index
FDG-PET/CT: 18F-fluorodeoxyglucose positron emission tomography
HR: hazard ratio
CT: computed thopmography
HIPEC: hyperthermic intraperitoneal chemotherapy
